# Editorial: Big data and artificial intelligence for genomics and therapeutics – Proceedings of the 19th Annual Meeting of the MidSouth Computational Biology and Bioinformatics Society (MCBIOS)

**DOI:** 10.3389/fbinf.2024.1470107

**Published:** 2024-08-09

**Authors:** Huixiao Hong, Inimary Toby-Ogundeji, Robert J. Doerksen, Zhaohui Steve Qin

**Affiliations:** ^1^ National Center for Toxicological Research, United States Food and Drug Administration, Jefferson, AR, United States; ^2^ Biology Department, University of Dallas, Irving, TX, United States; ^3^ Department of BioMolecular Sciences, University of Mississippi, University, MS, United States; ^4^ Department of Biostatistics and Bioinformatics, Emory University, Atlanta, GA, United States

**Keywords:** big data, bioinformatics, artificial intelligence, genomics, therapeutics

The 19th annual MidSouth Computational Biology and Bioinformatics Society (MCBIOS) conference took place on the University of Dallas campus over the span of three days (March 15th–17th, 2023). The conference theme was “*Big Data and Artificial Intelligence for Genomics and Therapeutics*”. The program consisted of five keynote sessions, 10 breakout scientific sessions (with 30 invited speakers) and four hands-on workshops. The conference focused on cutting-edge topics in bioinformatics and computational biology, including application of big data and machine learning in precision medicine, machine learning and deep learning in safety evaluation and risk assessment, network medicine and drug discovery, single-cell multi-omics analysis, and computational approaches for immuno-oncology. In addition, some 60 posters were presented by scientists and trainees at the conference, highlighting the interdisciplinary nature of bioinformatics and its critical role in advancing biomedical research and healthcare. This research topic collects six articles contributed from outstanding presentations at this conference, five of which appear in *Frontiers in Bioinformatics and one in Frontiers in Artificial Intelligence.*

Huang et al. developed PAGER-scFGA, a novel tool for single-cell functional genomics analysis aimed at understanding cellular responses to stress and disease. PAGER-scFGA integrates cell functional annotations and gene-set enrichment analysis into existing single-cell analysis pipelines like Scanpy, enabling the identification of cell functions through enrichment of potential cell-marker genesets. It provides pathways, annotated gene lists, and gene signatures enriched in specific cell subsets, aiding in the characterization of molecular mechanisms underlying cell trajectories. Through a case study on mouse natural killer cells, PAGER-scFGA unveils stages and trajectories of NK cell maturation, highlighting cell cytotoxicity and response to interleukin signaling pathways. Overall, PAGER-scFGA offers a comprehensive knowledge map of gene networks and functional compartments, expected to be a vital tool for inferring cell functions and detecting molecular mechanisms in single-cell studies. The web app is publicly available for further exploration.

High-throughput sequencing has greatly increased gene expression data, now accessible in repositories like NCBI’s GEO. Efficiently querying and analyzing this vast data, especially for artificial intelligence (AI)/machine learning (ML), is challenging. BioVDB addresses this by serving as a specialized vector database for gene expression data, using Automatic Label Extraction (ALE) to annotate samples with metadata like age, sex, and tissue type. Created by Winnicki et al., BioVDB includes 438,562 samples from eight microarray platforms, enhancing data retrieval with similarity search to identify patterns and infer missing labels. This feature supports rapid similarity analysis, crucial for uncovering biological phenomena. By integrating with AI/ML tools, BioVDB bridges the gap between large datasets and advanced computational analysis, fostering deeper insights and accelerating biological discovery.

The FDA Adverse Events Reporting System (FAERS) database is crucial for post-marketing drug safety reviews, but its effectiveness is hampered by inconsistent drug naming. This heterogeneity arises partly because the database includes both mandatory reports prepared by pharmaceutical companies and voluntary submissions from patients and healthcare professionals. Studies using FAERS without normalizing drug names can yield incomplete and inaccurate results. The study by Le et al. highlights the utility of RxNorm, a tool from the National Library of Medicine, for standardizing drug names in FAERS. By mapping prescription opioids to their RxNorm identifiers, the study demonstrated a significant reduction in name diversity, improving users’ ability to access information from the database accurately. With over 2,000 unique opioid names identified, RxNorm proved efficient in creating a uniform dataset. This method can enhance data quality in pharmacovigilance, offering a reliable foundation for diverse research applications.

The perspective by Patel et al. introduces the “No-Boundary Thinking” session on the Mid-South Computational Bioinformatics Society’s (MCBIOS) 19th annual meeting. No-boundary thinking fosters innovation by encouraging the scientific community to transcend traditional limitations and norms. This mindset allows for the discovery of new opportunities and the creation of groundbreaking solutions. The session highlighted this concept, particularly in the context of AI in bioinformatics. During the “No-Boundary Thinking” session, participants explored the future of AI in bioinformatics over the next 30 years. They discussed the integration of tools like ChatGPT to enhance bioinformatics research, facilitating communication among scientists from various disciplines to maximize the potential of AI algorithms. Additionally, the session emphasized the importance of educational outreach to inspire the next-generation of data scientists and informaticians. By embracing no-boundary thinking, the bioinformatics field can continue to evolve, driving forward with innovative and interdisciplinary approaches.

Type IV secretion systems (T4SSs) play a crucial role in the conjugation process of enteric bacteria, facilitating the transfer of plasmids that often contain antimicrobial resistance (AMR) genes. Algarni et al. developed a comprehensive plasmid transfer gene dataset, part of the FDA’s Virulence and Plasmid Transfer Factor Database, to analyze and compare conjugation-associated genes. By extracting relevant genes from GenBank, the study created tools to assess sequence diversity and compare plasmid transfer genes across different plasmid types. The plasmid transfer factor profile assessment and plasmid transfer factor comparison tools were instrumental in evaluating plasmids from GenBank and whole genome sequencing data. The findings demonstrated that these tools significantly enhance our understanding of how T4SSs and conjugative plasmids contribute to AMR gene dissemination, providing valuable insights for combating antimicrobial resistance.

Recent advances in deep learning have significantly improved contact map-based protein 3D structure prediction. Despite this, accessible software tools for beginners remain scarce. Baker et al. introduced GoFold, a user-friendly graphical user interface designed to simplify the contact map overlap (CMO) problem for novice users, aiding in better template selection. GoFold distinguishes itself with its intuitive design and thorough tutorials, making it accessible to those without extensive prior knowledge. It allows users to input proteins in various formats and visualize CMO’s to aid understanding of which overlaps are problematic. The authors compared GoFold’s capabilities to those of the state-of-the-art method, map_align, using PSICOV and CAMEO datasets, and showed GoFold’s superior performance for prediction of the correct protein fold and for alignment of target protein to template. Running efficiently on personal computers without third-party dependencies, GoFold is freely available for macOS, Linux, and Windows, promoting broad accessibility.

The papers included in this Research Topic provide examples of big data and artificial intelligence for genomics and therapeutics. They demonstrate the excellent studies from MCBIOS members in applying machine learning methods to extract valuable insights from big data.

